# Do we really know who has an *MGMT* methylated glioma? Results of an international survey regarding use of *MGMT* analyses for glioma

**DOI:** 10.1093/nop/npz039

**Published:** 2019-09-24

**Authors:** Annika Malmström, Małgorzata Łysiak, Bjarne Winther Kristensen, Elizabeth Hovey, Roger Henriksson, Peter Söderkvist

**Affiliations:** 1 Department of Advanced Home Care, Linköping University, Sweden; 2 Department of Clinical and Experimental Medicine, Linköping University, Sweden; 3 Department of Pathology, Odense University Hospital, Institute of Clinical Research, University of Southern Denmark; 4 Department of Medical Oncology, Nelune Comprehensive Cancer Centre, Prince of Wales Hospital, Randwick, Sydney, NSW, Australia; 5 University of New South Wales, Sydney, Australia; 6 Department of Radiation Sciences, University of Umeå, Sweden

**Keywords:** glioma, international consensus guidelines, international survey, laboratory methods and cutoff level, MGMT testing

## Abstract

**Background:**

Glioma O6-methylguanine-DNA methyltransferase (*MGMT*) promoter methylation status informs clinical decision making. Worldwide different methods and cutoff levels are used, which can lead to discordant methylation results.

**Methods:**

We conducted an international survey to clarify which methods are regularly used and why. We also explored opinions regarding international consensus on methods and cutoff.

**Results:**

The survey had 152 respondents from 25 countries. *MGMT* methylation status is determined for all glioblastomas in 37% of laboratories. The most common methods are methylation-specific polymerase chain reaction (msPCR) (37%) and pyrosequencing (34%). A method is selected for simplicity (56%), cost-effectiveness (50%), and reproducibility of results (52%). For sequencing, the number of CpG sites analyzed varies from 1–3 up to more than 16. For 50% of laboratories, the company producing the kit determines which CpG sites are examined, whereas 33% select the sites themselves. Selection of cutoff is equally distributed among a cutoff defined in the literature, by the local laboratory, or by the outside laboratory performing the analysis. This cutoff varies, reported from 1% to 30%, and in 1 laboratory tumor is determined as methylated in case of 1 methylated CpG site of 17 analyzed. Some report tumors as unmethylated or weakly vs highly methylated. An international consensus on *MGMT* methylation method and cutoff is warranted by 66% and 76% of respondents, respectively. The method preferred would be msPCR (45%) or pyrosequencing (42%), whereas 18% suggest next-generation sequencing.

**Conclusion:**

Although analysis of *MGMT* methylation status is routine, there is controversy regarding laboratory methods and cutoff level. Most respondents favor development of international consensus guidelines.

For more than a decade, temozolomide (TMZ) and other alkylating agents have been key components of glioma treatment.^1–4^ Their efficacy has been demonstrated to be dependent on the methylation status of the O6-methylguanine-DNA methyltransferase (*MGMT*) promoter in tumor tissue, where only those with methylated promoter are expected to meaningfully respond to alkylating agent treatment.^5–7^ For elderly patients with glioblastoma (GBM), not deemed fit for combined treatment both with radiotherapy and TMZ, the European Association of Neuro-Oncology has recommended that *MGMT* status of the tumor be included in the treatment decision process, according to findings in 2 pivotal trials.^8–10^ Several methods of analyzing *MGMT* promoter methylation status have been published, and different cutoffs for defining a methylated status have been reported for individual methods.^11–24^ It is considered that the majority of tumors can be reliably classified as either *MGMT* promoter methylated or unmethylated, but the use of different methods and/or cutoff values will lead to discordant results in some patients, thereby potentially jeopardizing optimal treatment recommendations.^25^ This is particularly true for tumors displaying only weak or focal (borderline) methylation at the investigated CpG sites of the *MGMT*–associated 5’-CpG island (gray-zone tumors). Of further concern, a study by Lassman et al analyzing concordance between locally and centrally analyzed *MGMT* found discordant results in 39% of analyzed samples.^26^

We conducted an international survey to find out which analyses and cutoff levels are currently used in different clinicopathological settings and canvassed opinions regarding the importance of developing international consensus guidelines.

## Methods

A survey with 27 questions was sent electronically to national and international neuropathology societies (see Acknowledgments) as well as to scientists engaged in *MGMT* testing of glioma. The societies forwarded the survey to their members. Responses were collected between January 31, 2018, and July 18, 2018. The survey focused on identifying which patient groups generally have tumor tissue analyzed and provision of a given reason for choosing a specific method and cutoff level. We also enquired about access to, frequency of, and financing of *MGMT* testing. We asked for comments and recommendations regarding *MGMT* testing and opinions with respect to the development of international consensus guidelines. For the survey questions, see Supplementary Table S1.

## Results

The survey was answered by 152 respondents—mainly neuropathologists—from 25 countries, the majority from North America, followed by Europe (See Table 1).

**Table 1:** Origin of Respondents

Region and Country	Number of Responses
North America	71
United States of America	65
Canada	6
South America	2
Argentina	1
Brazil	1
Europe	44
Germany	9
Sweden	5
United Kingdom	5
Spain	4
Denmark	3
Finland	3
Norway	3
Switzerland	3
The Netherlands	2
Austria	1
Hungary	1
Israel	1
Lithuania	1
Poland	1
Slovenia	1
Turkey	1
Australasia	16
Japan	12
Hong Kong	1
Australia	1
Korea	1
New Zealand	1
Not reported	19

### *Availability of* MGMT *Testing*

**Fig. 1:**
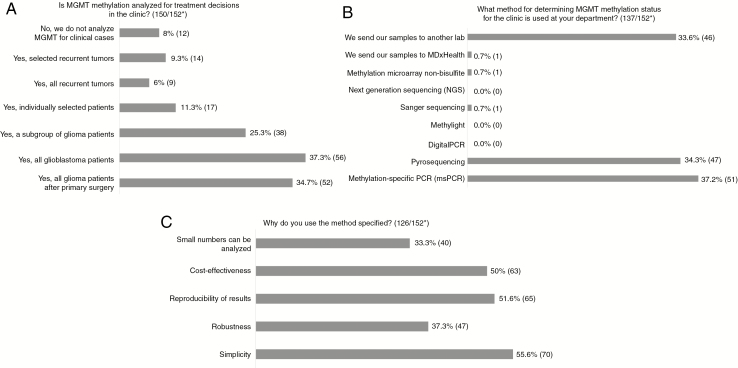
A-C, Results of Questions Regarding O6-Methylguanine-DNA Methyltransferase (*MGMT*) Analysis *Number of answers/total number of respondents to the survey. For some questions more than one alternative can be chosen; therefore, total can be more than 100%.

*MGMT* methylation status is determined for all GBMs in 37% of laboratories, whereas 35% analyze tissue from all gliomas (also lower grades) at the time of primary surgery. Subgroups of patients having tumor tissue analyzed are elderly, (defined as older than 60, 65, 70, or 75 years, depending on the center), all high-grade glioma, including grade 3, or only those requested by the neurosurgeon or oncologist. Some laboratories routinely analyze all recurrent tumors, others study *MGMT* status only in selected cases. *MGMT* analysis is not performed by 8% of respondents’ laboratories (Fig. 1A).

### Methods

The most commonly used methods are bisulfite methylation-specific polymerase chain reaction (msPCR) (37%) and pyrosequencing (34%), whereas 34% send their samples to an outside laboratory (method of outside laboratory not requested within the survey) (Fig. 1B).

Reasons for selecting msPCR are stated as: “According to clinician, best correlation with clinical outcome” and turnaround time. For msPCR qualitative and quantitative methodologies are both reported.

Reasons provided for selection of pyrosequencing include its high specificity, that it is objective, that “Pyrosequencing can quantify methylation at each CpG and therefore is a truly quantitative method” and has “the best predictive value for response to TMZ in comparison to other methods, according to the literature”.^17^

A number of additional methods are reported to be in use, such as Sanger sequencing, melting curve analysis, Illumina 450 k or 850 k methylation (Methyl EPIC) analysis together with or without the DKFZ classifier (https://www.molecularneuropathology.org/mnp) all following bisulfite treatment, methylation-specific multiplex ligation-dependent probe amplification (MS-MLPA), immunohistochemistry (IHC), and *MGMT* mRNA absolute value analyzed by real-time reverse transcriptase (RT)-PCR.

The 3 main reasons cited by respondents for selecting a specific method are simplicity (56%), reproducibility of results (52%), and cost-effectiveness (50%) (Fig. 1C). One respondent’s comment on choice of method was: “We compared msPCR, single-nucleotide primer extension (SNuPE), combined bisulfite restriction analysis (COBRA), and pyrosequencing and for formalin-fixed paraffin embedded tissue (FFPE) pyrosequencing was clearly superior especially with reproducibility.” Motives cited for using an alternative method for msPCR or pyrosequencing were simplicity and cost-effectiveness (melting curve analysis) and “decreased risk of PCR contamination” (methylation-specific restriction endonuclease followed by quantitative PCR). One laboratory reported using real-time RT-PCR because “We have the evidence of *MGMT* mRNA amount and TMZ sensitivity” together with robustness, reproducibility of results, and the fact that small numbers can be analyzed. Some sites use pyrosequencing and, in parallel, Illumina Infinium EPIC arrays, claiming robustness, reproducibility of results and cost-effectiveness, together with this providing methylome data as well. Reported reasons to use IHC include it being “easy and fast,” low costs, “results are reproducible,” and small numbers can be analyzed.

**Fig. 2:**
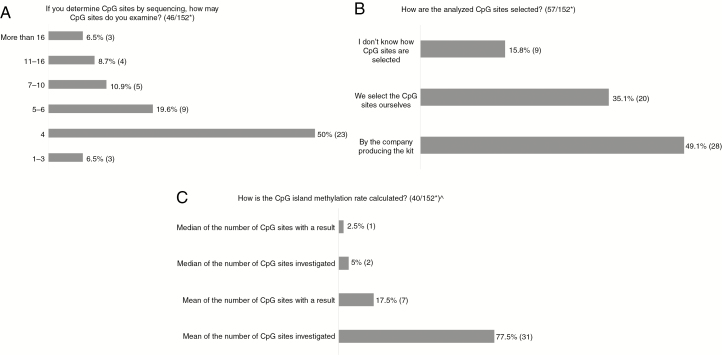
A-C, Results of Questions Regarding CpG Sites Examined *Number of answers/total number of respondents to the survey. ^One respondent answered “Median of number of CpG sites investigated” as well as “Mean of number of CpG sites investigated.”

For those using sequencing, 50% analyze 4 CpG sites, but the number varies between 1–3 and more than 16 (Fig. 2A). For half the laboratories, the company producing the kit determines which CpG sites are examined, whereas one-third select the sites themselves (Fig. 2B). The majority define methylation rate as the mean for the number of CpG sites investigated, whereas some use median and others include CpG sites only where a result for methylation is obtained (Fig. 2C).

### Cutoff Levels

**Fig. 3:**
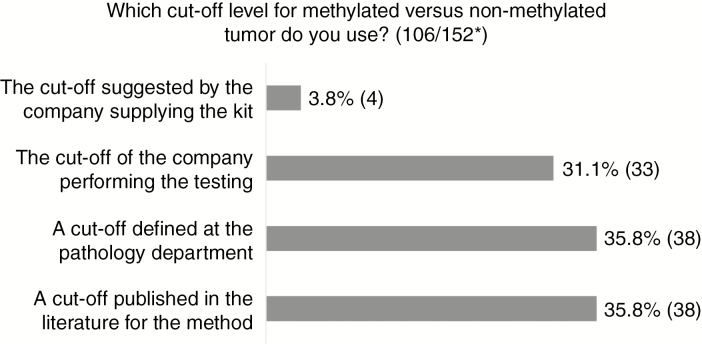
Results of Question Regarding Cutoff for O6-Methylguanine-DNA Methyltransferase (*MGMT*) Methylation *Number of answers/total number of respondents to the survey.

Cutoff values for the different methods are equally distributed between a cutoff a) published in the literature, b) defined by the local laboratory, or c) defined by the outside laboratory or company performing the analysis. Only 4% use a cutoff value suggested by the company supplying the kit (Fig. 3). For those that use a cutoff of 9% or more or 10% or more methylated alleles for methylated *MGMT*, the majority use pyrosequencing or msPCR technique. Forty-seven percent use another cutoff value, varying among different pathology departments and being partly method dependent.

For qualitative msPCR, some report on methylated band in the gel electrophoresis being present or not, or “Anything which looks >20% by band intensity is reported as methylated.”

For quantitative msPCR, a methylation score of 2.0 or greater is used by some (often calculated as the copy number of methylated *MGMT* normalized to the β-actin gene^8,27^), whereas others report number of methylated sites, and still others consider 1% or more methylated alleles in adequate tumor tissue as methylated.

For pyrosequencing, some institutions report the use of a cutoff of 30% methylated alleles, whereas others regard more than 3% methylated alleles as a methylated tumor. Other cutoff levels for methylated vs unmethylated tumors are more than 2 out of 4 CpG sites being more than 10% methylated, or more than 10% in 1 of 4 sites. For 1 respondent using Sanger sequencing, a tumor is defined as methylated if there is 1 methylated CpG site out of 17 analyzed.

For msPCR and pyrosequencing, several laboratories subdivide the results of *MGMT* promoter methylation into more than 2 groups, such as “unmethylated” vs “weakly methylated” vs “strongly methylated”.

### Testing Frequency

The number of *MGMT* tests performed by individual laboratories varies from daily runs to a couple of runs per year, with the majority being once per week (44%) or 2 to 4 times per week (38%). The number of samples analyzed in a run is reported to be between 1 and 24.

### Financing of Testing

In most countries the pathology, neurosurgery, and/or oncology/radiotherapy department pay for the analyses. In some of these countries health care is financed by taxes, otherwise by the patient’s health insurance. The test is billed, at least in part, to insurance companies in the United States, Germany, Argentina, Spain, Switzerland, Slovenia, and Israel. The patient can be requested to pay an extra sum specifically for the *MGMT* testing in the United States, Australia, Spain, Turkey, Israel, Japan, Korea, Poland, and Germany.

### International Consensus

**Fig. 4:**
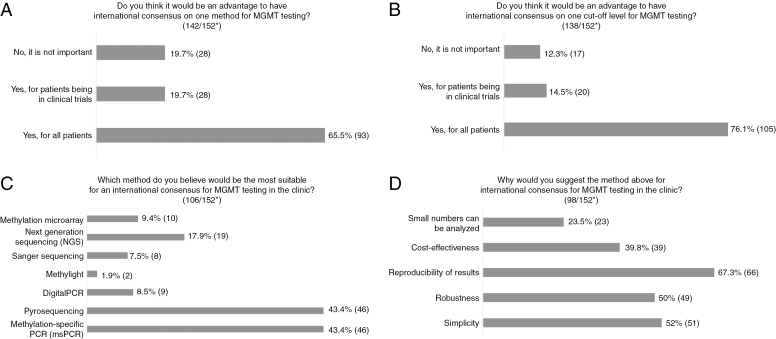
A-D, Results of Questions Regarding International Consensus for O6-Methylguanine-DNA Methyltransferase (*MGMT*) Testing *Number of answers/total number of respondents to the survey. For some questions more than one alternative can be chosen; therefore, total can be more than 100%

An international consensus on *MGMT* promoter methylation method for all patients is believed to be of advantage by 65.5%, whereas 1 in 5 respondents do not find a consensus necessary (Fig. 4A). A consensus on a cutoff level is warranted by 76% (Fig. 4B). Most suggest that the consensus method should be msPCR or pyrosequencing, whereas 18% suggest next-generation sequencing (NGS) (Fig. 4C). The most important reason provided to select a specific testing method is reproducibility of results (Fig. 4D).

### Comments From Respondents

A number of issues are raised by the respondents. Any recommended method should preferably be “fast, affordable, reproducible, and scalable to high throughput.” Respondents cite the need for provision of more evidence for methods for which individual CpG sites are analyzed regarding which sites should be investigated, the significance of the percentage of methylation at each site, and of increased methylation at a limited number of CpG sites. The necessary tumor cell content in the analyzed tissue for correct assessment of tumor *MGMT* methylation status needs to be addressed. A high proportion of normal brain cells in the analyzed sample might affect the *MGMT* methylation result. Turnaround time for tumor analysis might influence choice of method. For instance, the Methylation EPIC BeadChip (850 k array) will often take 2 weeks to obtain results, which may be too long for clinicians. Respondents also reported that the cost for testing is important in informing their choice. Availability of equipment for testing will influence the method used at the laboratory. An international consensus on method(s) might lead to an outcome that some laboratories no longer can perform *MGMT* testing.

Advantages with international guidelines, according to the respondents, would be that “consensus would establish consistency” and “a consensus on cutoff could help neuro-oncologists in treatment recommendations, instead of treating patients based on any percentage of methylation.” Also, in the scenario of establishment of an international consensus, “the negligence regarding the availability of testing might change” in countries where the test is not currently available.

Whereas some feel that “There is an urgent need for both standardization and validation of the method used for (*MGMT* promoter methylation) testing,” a small number of respondents question the relevance of this both for biological and clinical reasons, as they believe that “Too much undue emphasis is being placed on *MGMT* methylation status. Life is more complex than the methylation pattern of a single gene” and that “the consequences of misclassification (methylated rather than unmethylated, or vice versa) are rarely significant.”

The respondents propose several methods for increasing congruence in determining *MGMT* methylation status, such as consensus on using quantitative assays, but not just one. They request method-dependent international guidelines that ideally should include technical recommendations along with guidance on interpretation of results. Apart from continued use of msPCR or pyrosequencing, some respondents believe it would be an advantage to add *MGMT* testing to NGS panel sequencing, or to determine *MGMT* status as part of performing a methylation array or to use “any other method that is appropriately validated.” Some respondents suggest that international proficiency programs (in *MGMT*-methylation assessment techniques) be developed. They point out that it is important that the “future development of better methods is not hampered by the establishment of guidelines.”

One respondent requests a consensus on how to handle cases showing borderline (gray-zone) methylation levels, and proposes testing with 2 alternative methods. Harmonization of test results by interlaboratory control programs, national as well as international, are recommended. Consensus on the cutoff for each method should be achieved and compared with cutoffs between methods using the same set of samples. Alternatively, “if criteria for determining a cutoff level were defined, the actual cutoff could vary between laboratories, but be uniform in meaning.”

## Discussion

*MGMT* promoter methylation status is currently the strongest treatment-predictive factor for response to alkylating agent therapy for patients with GBM.^6,8,9,27,28^ Several methods and cutoffs are available for *MGMT* analyses.^11–24^ The need for standardization of *MGMT* testing was already addressed in 2010, recommending comparison of different techniques across different laboratories and validation of cutoff levels.^29^ In a survey from 2016 regarding the use of testing of molecular markers in neuropathology, *MGMT* testing was rated as the most problematic.^30^ The results of our survey clearly indicate that *MGMT* methylation status has been incorporated into clinical decision making. The diversity of methods and cutoff levels used worldwide confirms and reiterates the concerns previously raised. A number of publications have addressed the challenges regarding *MGMT* analyses.^13,15,16,24,25,31^ In a recent review Mansouri et al describe several of the methodologies reported in this survey, together with pros and cons, and provide recommendations on how to proceed in trials and clinical settings.^32^

The promoter region of *MGMT* displays a heterogeneous methylation pattern, and the different CpG sites have variable clinical significance for *MGMT* gene silencing.^33–36^ The choice of CpG sites to examine will therefore be of critical importance for the clinically correct assessment of methylation status. Malley and colleagues found the differentially methylated regions DMR1 and DMR2 as the most influential for transcriptional silencing. These are positioned between –232 base pair (bp) to –63 bp (DMR1) and from +106 bp to +225 bp (DMR2), respectively, from the transcriptional start site (TSS).^35^ Several additional studies have confirmed 2 CpG clusters, 1 upstream and 1 downstream from the TSS, as being crucial. These CpG-rich regions are more or less overlapping among the different investigations.^33–36^

An explanation underlying the principal problem of defining reliable cutoffs across different methods and laboratories is due to the high degree of heterogeneity in CpG site methylation from tumor to tumor and the still-limited knowledge on the degree and pattern of methylation that is minimally required to show a clinical effect in terms of response to TMZ and prognosis. This biological heterogeneity is further complicated by the heterogeneity of tissue samples available for molecular testing, for example, with respect to tumor cell content. The latter issue might be solved to a degree by selection of tissue for testing from different parts of the tumor and microdissection of cellular tumor areas before DNA extraction.

Apart from the biological factors, comparisons among different methods published in the literature can be difficult because of the different CpG sites analyzed, different cutoff values also for the same method, heterogeneous tumor samples, lack of quality assurance of the method, and lack of validation of results in independent cohorts.^32^ In view of the widespread discordant results between locally and centrally analyzed tumors,^26^ for correct interpretation of the results of clinical trials it seems essential that central *MGMT* analyses with one method be performed. For trials focusing on patients with either methylated or unmethylated tumors, this will be crucial so the intended patient population is studied and potentially effective treatment is not withheld.

MsPCR has been validated in several large randomized trials, such as the Nordic trial, the NOA-08, and the Canadian National Cancer Institute of Canada (NCIC) elderly trial,^8,9,27^ confirming the role of *MGMT* methylation for response to TMZ at the group level. In the NCIC trial^27^ those patients with an *MGMT* methylated tumor randomly assigned both to radiotherapy and TMZ had the best survival. However, in addition, the subgroup analyses for those deemed as having an unmethylated tumor, comparing radiotherapy alone or in combination with TMZ showed a statistically nonsignificant trend toward increased survival for the combination (median survival for radiotherapy 7.9 months vs 10.0 months for chemoradiation). These results have been used as proof of efficacy of TMZ also for GBM patients with unmethylated *MGMT*.^37^ Of importance, there was no difference in progression-free survival between the 2 treatment arms, indicating that any survival benefit in the combination arm would be due to treatment received after TMZ, at first progression. An alternative explanation could be that the cutoff level used was not optimal. This idea is supported by a study by Dovek et al, in which the commonly used cutoff of 2 or greater (see *Cutoff Levels* for explanation) has been shown not to subdivide patients as expected. They found that a fraction of patients deemed as having an unmethylated tumor (score 1 to 1.99) had survival similar to those considered to be methylated (score ≥2), whereas those with a score less than 1 had poorer outcome.^38^ This is confirmed in a recent publication indicating that patients with *MGMT* status determined by msPCR with results between clearly methylated and unmethylated (gray zone) seem to benefit from TMZ treatment.^39^

Pyrosequencing seems to have gained support especially because of the study by Quillien and colleagues,^17^ as several respondents refer to this publication when advocating for this method. They compared 5 different methods of *MGMT* analysis: MsPCR, MethyLight, pyrosequencing, methylation-sensitive high-resolution melting, and IHC. The cohort consisted of 100 patients treated with concomitant radiochemotherapy with TMZ. For both progression-free and overall survival, pyrosequencing had the best predictive value, together with good reproducibility and high sensitivity. Several other trials also seem to support the use of pyrosequencing.^16,19,40^

MS-MLPA has the advantage that no bisulfite conversion of DNA is necessary. However, with this approach, the CpG sites that can be selected for analysis are limited, as they must contain the HhaI restriction site GCGC. Christians et al analyzed *MGMT* status for 35 patients from a phase 2 trial comparing msPCR, pyrosequencing, and MS-MLPA. They concluded that pyrosequencing was suitable for high-throughput settings and msPCR for clinical routine diagnostics with low sample numbers. MS-MLPA was not recommended because of inferior results.^41^

The Infinium Methyl EPIC methylation array was suggested by some respondents. The *MGMT* gene region including the promoter is well covered and focuses on DMR1 and DMR2. In a study by Bady et al,^36^*MGMT*-STP27 was shown to correlate to survival in TMZ-treated patients. The method is useful for clinical samples, as it yields reliable results for FFPE tissue also.^42^ The cost per sample is high, however, and turnaround time can be too long for clinical cases.

Combined analyses with 2 different methods are proposed as a possible way to handle tumors with unclear methylation status when analyzed by 1 method.^40,43,44^ In a study by Dahlrot and colleagues, *MGMT* testing by pyrosequencing was combined with IHC analysis for detection of MGMT protein in tumor cells only. By excluding normal cells, the results of tumor *MGMT* methylation were not confounded. This combined approach improved the prediction of response to TMZ treatment.^45^

Some respondents questioned the clinical implications of *MGMT* analyses. In the clinical scenario of elderly patients with truly unmethylated *MGMT*, if these patients receive TMZ as monotherapy, they are likely to rapidly progress.^8,9^ This could in many cases make a switch to radiotherapy later impossible because of deterioration in performance status. A correct upfront treatment choice is crucial for survival, as was indicated both in the Nordic and NOA-08 trials.^8,9^ In these trials, those patients randomly assigned to TMZ monotherapy and having an unmethylated tumor seem to have the poorest survival, also compared with radiotherapy alone. Apart from the risk of causing inferior survival, though generally considered to have low toxicity, TMZ can cause life-threatening side effects.^28,46^ Treating patients with chemotherapy highly likely to be ineffective can justly be questioned, and is a needless expense for countries with limited health care resources.^47^

There are a number of publications addressing the existing difficulties with *MGMT* testing and recommending pragmatic ways to cope with this challenge in the clinical setting.^14–16,21,24,25,32,40,41,48^ Despite this, evidently those working in the field and answering this survey still feel that this is not sufficient. Most respondents suggest that an international consensus both on method and cutoff should be established. There is a need to define and recommend the current best methods and the clinically relevant cutoff for methylated tumors. The respondents request that the recommended reference method(s) in such a consensus guideline have existing supportive evidence in the literature. This technique (or these techniques) should be able to select patients with the greatest chance of responding to alkylating agent therapy. There should also be possibilities for harmonization of test results by interlaboratory control programs. Reanalysis of randomized trials with *MGMT* status determined by one method, by an additional technique, might be helpful by providing comparisons among different analytical methods in defined patient populations.

Another important issue, the necessary proportion or volume of tumor cell content in the analyzed sample, needs to be resolved before it will be possible to establish an accurate and reproducible cutoff for *MGMT* testing.

For methods focusing on CpG site analyses, apart from defining which CpG sites should be evaluated and how, there is a need to define how methylation status should be calculated from the results obtained. Further, guidelines for evaluation of new emerging methods would be valuable.

The results from this survey confirm the urgent need for international collaboration regarding *MGMT* analyses in the clinical setting. The aim should be to define the clinically most accurate methods. These methods should be quantitative and probably should include analyses of DMR1 and DMR2. For this msPCR and pyrosequencing techniques are both currently suitable. An international expert group could define what additional validation of the clinically relevant cutoffs for methylated vs unmethylated tumors for response to alkylating treatment is needed. They could additionally suggest further steps to be able to reach consensus.

Consensus on *MGMT* testing would increase the validity of informed decision making regarding treatment with TMZ and other alkylating agents in the everyday care of patients with glioblastoma.

## Supplementary material

Supplementary material is available online at *Neuro-Oncology* (http://neuro-oncology.oxfordjournals.org/).

npz039_suppl_Supplementary_Table
