# Supplementary material for: Do we really know who has an MGMT methylated glioma? Results of an international survey regarding use of MGMT analyses for glioma
Source: Neurooncol Pract. 2019 Sep 24;7(1):68–76. doi: 10.1093/nop/npz039 (PMC6993038; doi:10.1093/nop/npz039)
Supplement: npz039_suppl_Supplementary_Table [file npz039_suppl_supplementary_table.docx]

| **Table S1. International survey regarding use of MGMT analyses for glioma** | | |  |  |  |  |  |  |  |
| --- | --- | --- | --- | --- | --- | --- | --- | --- | --- |
| 1. Is MGMT methylation analyzed for treatment decisions in the clinic? | Yes, all glioma patients after primary surgery | Yes, all glioblastoma patients | Yes, a subgroup of glioma patients | Yes, individually selected patients | Yes, all recurrent tumors | Yes, selected recurrent tumors | No, we do not analyze MGMT for clinical cases |  |  |
| 2. If MGMT is analyzed for another setting than above (question 1), please note below which patients are selected |  |  |  |  |  |  |  |  |  |
| 3. What method for determining MGMT methylation status for the clinic is used at your department? | Methylation-specific PCR (msPCR) | Pyrosequencing | Digital PCR | Methylight | Sanger sequencing | Next-generation sequencing (NGS) | Methylation microarray (non-bisulfite treated) | We send our samples to MDxHealth for MGMT testing | We send our samples to another laboratory |
| 4. If another method or laboratory is used for determining MGMT methylation status (question 3), please note below which method or lab is selected |  |  |  |  |  |  |  |  |  |
| 5. Why do you use the method specified above (question 3 and 4)? | Simplicity | Robustness | Reproducibility of results | Cost-effectiveness | Small numbers can be analyzed |  |  |  |  |
| 6. If another or additional reason, please note below |  |  |  |  |  |  |  |  |  |
| 7. If you determine CpG sites by sequencing, please answer the following questions, otherwise move to question number 10. How many CpG sites do you examine? | 1-3 | 4 | 5-6 | 7-10 | 11-16 | more than 16 |  |  |  |
| 8. How are the analyzed CpG sites selected? | They are selected by the company producing the kit | We select the CpG sites ourselves | I don´t know how CpG sites are selected |  |  |  |  |  |  |
| 9. How is the CpG island methylation rate calculated? | Mean of the number of CpG sites investigated | Mean of the number of CpG sites where a result is obtained | Median of the number of CpG sites investigated | Median of the number of CpG sites where a result is obtained |  |  |  |  |  |
| 10. Which cut-off level for methylated versus non-methylated tumor do you use? | A cut-off published in the literature for the method | A cut-off defined at the pathology department | The cut-off of the company performing the testing | The cut-off suggested by the company supplying the kit |  |  |  |  |  |
| 11. If another method for defining the cut-off level is used, please note the method below |  |  |  |  |  |  |  |  |  |
| 12. What cut-off level do you use for defining methylated versus unmethylated tumor? | <9% | <10% | Another cut-off level |  |  |  |  |  |  |
| 13. If you use another cut-off level for methylated versus unmethylated tumor, please note cut-off level below |  |  |  |  |  |  |  |  |  |
| 14. Who pays for the testing? | The pathology department | The oncology/radiotherapy department | An insurance company | The patient |  |  |  |  |  |
| 15. If testing is paid by other means, please note below |  |  |  |  |  |  |  |  |  |
| 16. How often is MGMT testing performed, in average? | Daily, working days (5 or more times per week) | 2-4 times per week | 1 time per week | Every 2 weeks | When a number of cases have been collected |  |  |  |  |
| 17. Please comment on the frequency of MGMT testing, if none of the above apply |  |  |  |  |  |  |  |  |  |
| 18. Please comment on the number of samples you analyze in a run |  |  |  |  |  |  |  |  |  |
| 19. Do you think it would be an advantage to have international consensus on one method for MGMT testing? | Yes, for all patients | Yes, for patients being in clinical trials | No, it is not important |  |  |  |  |  |  |
| 20. Do you think it would be an advantage to have international consensus on one cut-off level for MGMT testing? | Yes, for all patients | Yes, for patients being in clinical trials | No, it is not important |  |  |  |  |  |  |
| 21. Do you have any additional comment regarding the above questions? |  |  |  |  |  |  |  |  |  |
| 22. Which method do you believe would be the most suitable for an international consensus for MGMT testing in the clinic? | Methylation-specific PCR (msPCR) | Pyrosequencing | Digital PCR | Methylight | Sanger sequencing | Next generation sequencing (NGS) | Methylation microarray |  |  |
| 23. If you suggest another method for international consensus for MGMT analysis, please note the method below |  |  |  |  |  |  |  |  |  |
| 24. Why would you suggest the method above for international consensus for MGMT testing in the clinic? | Simplicity | Robustness | Reproducibility of results | Cost-effectiveness | Small numbers can be analyzed |  |  |  |  |
| 25. Are there any additional reasons to choose the method you prefer? |  |  |  |  |  |  |  |  |  |
| 26. Do you have any further comments? |  |  |  |  |  |  |  |  |  |
| 27. In which country are you working? |  |  |  |  |  |  |  |  |  |
| Contact information (voluntary information) |  |  |  |  |  |  |  |  |  |
|  |  |  |  |  |  |  |  |  |  |
